# Plasma sex hormone-binding globulin predicts neurodegeneration and clinical progression in prodromal Alzheimer's disease

**DOI:** 10.18632/aging.103497

**Published:** 2020-07-22

**Authors:** Wei Xu, Bing-Jie Su, Xue-Ning Shen, Yan-Lin Bi, Chen-Chen Tan, Jie-Qiong Li, Xi-Peng Cao, Qiang Dong, Lan Tan, Alzheimer’s Disease Neuroimaging Initiative, Jin-Tai Yu

**Affiliations:** 1 Department of Neurology, Qingdao Municipal Hospital, Qingdao University, Qingdao, China; 2 Department of Neurology and Institute of Neurology, Huashan Hospital, Shanghai Medical College, Fudan University, Shanghai, China; 3 Department of Anesthesiology, Qingdao Municipal Hospital, Qingdao University, Qingdao, China; 4 Department of Neurology, The Affiliated Hospital of Qingdao University, Qingdao, China; 5 Clinical Research Center, Qingdao Municipal Hospital, Qingdao, China

**Keywords:** sex hormone-binding globulin, cerebrospinal fluid, plasma, Alzheimer’s disease, biomarker

## Abstract

It was unclear whether sex hormone-binding globulin (SHBG) was a circulating biomarker of Alzheimer’s disease (AD). We tested the cross-sectional relationships between plasma SHBG and cerebrospinal fluid (CSF) AD biomarkers in 707 non-demented adults. Next, the influences of plasma SHBG on dynamic changes of CSF Aβ42, hippocampus volume, brain metabolism, and cognition were explored in 448 non-demented adults from the Alzheimer’s disease Neuroimaging Initiative (ADNI). Finally, the predictive and diagnostic values of plasma SHBG in AD were explored. A positive correlation was found between SHBG levels in plasma and CSF. Individuals with higher plasma SHBG levels had lower CSF Aβ42 (p < 0.005), after adjusting for age, gender, education, *APOE*4 allele, and cognitive scores. Though no significant difference of plasma SHBG was observed between mild AD dementia and healthy normal, plasma SHBG could contribute to accelerated rates of CSF Aβ42 decrease (p < 0.0005), decline in brain metabolism (p < 0.05), and hippocampus atrophy (p < 0.01), cognitive decline (p < 0.01), as well as higher risk of AD dementia (p < 0.05). These findings indicated plasma SHBG could be a prodromal biomarker to predict disease progression in AD.

## INTRODUCTION

Sex hormone-binding globulin (SHBG) in plasma was significantly elevated in subjects with Alzheimer’s disease (AD) than their matched controls [1], suggesting SHBG might be a circulating biomarker of AD. In the past decade, several, but far from enough studies have been devoted to understanding the relationship between SHBG and AD. Most researchers focused on their cross-sectional relationships but the conclusions were disputable. This was possibly due to varying sources of biases, such as ascertainment bias due to AD diagnosis in the absence of pathological evidence [1–5]. Similarly, their longitudinal associations were also inconsistently reported [2, 6, 7]. It is a challenge to identify a circulating molecule as a diagnostic or predictive biomarker of AD, especially considering the complexity of the disease. AD is a continuous process underpinned by multiple prodromal biological alterations, including dysfunctional metabolism of cerebrospinal fluid (CSF) pathological proteins (lowered β-amyloid_1-42_ (Aβ42) and elevated tau proteins), functional and structural brain abnormalities, and finally the devastating cognitive impairments and social disability. Most changes could be seen in the prodromal stage of the disease, decades before the occurrence of AD. Thus, understanding the relationships with abnormalities of AD phenotypes will shed light on the predictive values of SHBG as an AD biomarker.

Herein, we aimed to investigate whether plasma SHBG could predict neurodegeneration and clinical progression in prodromal AD: 1) we first tested whether plasma SHBG was associated with CSF AD biomarkers in Chinese Alzheimer’s Biomarker and LifestylE (CABLE) study; 2) we next explored the values of plasma SHBG in predicting CSF AD core biomarkers, imaging, cognition, and AD risk in Alzheimer’s disease Neuroimaging Initiative (ADNI) cohort. Also, we tested whether plasma SHBG could be a good diagnostic biomarker of AD.

## RESULTS

### Characteristics of participants

A total of 707 non-demented individuals were derived from the CABLE. The mean (SD) age of the study sample was 62.5 (SD = 10.5) years old and females accounted for roughly 59%. Participants who had higher plasma SHBG levels tended to be at an older age (p < 0.0005) and with lower MMSE scores (p < 0.05). As for the ADNI, 448 non-demented individuals (non-Hispanic whites accounting for 93.3%) were included at baseline. The study sample was at an older age (74.8 ± 7.2 years) with a smaller female proportion (37%). Participants who had higher plasma SHBG levels tended to be older (p = 0.03) and female (p < 0.0001) (Table 1).

**Table 1:** Baseline characteristics of participants.

Cohort ^*^	**CABLE**				**ADNI**			
	**Total**	**Low**	**High**	***p* value**	**Total**	**Low**	**High**	***p* value**
N.	707	353	354	…	448	224	224	…
Age (mean ± SD, year)	62.5 ± 10.5	61.1 ± 10.4	64.0 ± 10.4	**0.0003**	74.8 ± 7.2	74.1 ± 7.2	75.6 ± 7.2	**0.038**
Gender (M/F)	291/416	133/220	158/196	0.06	282/166	168/56	114/110	**< 0.0001**
Education (mean ± SD, year)	9.7 ± 5.5	9.9 ± 4.4	9.6 ± 6.4	0.10	15.6 ± 3.0	15.6 ± 3.1	15.7 ± 2.9	0.93
*APOE*Ɛ4 carrier status (%)	16.5% (117)	15.9% (56)	17.2% (61)	0.62	47.5% (213)	48.2% (108)	46.9% (105)	0.78
MMSE (mean ± SD)	27.1 ± 3.2	27.3 ± 3.2	27.0 ± 3.1	**0.046**	27.3 ± 1.8	27.4 ± 1.8	27.1 ± 1.8	0.11
SHBG (mean ± SD, nmol/L)	52.9 ± 31.3	29.8 ± 10.2	75.9 ± 28.1	**< 0.0001**	61.7 ± 27.2	41.2 ± 10.2	82.3 ± 23.0	**< 0.0001**

MMSE: mini-mental state examination; SHBG: sex hormone-binding globulin; M: male; F: female

*cutoff is defined as median level of plasma SHBG: > 46.74 nmol/L for CABLE and > 56.23 nmol/L for ADNI

### Correlation analysis of SHBG levels in plasma and CSF

**Figure 1:**
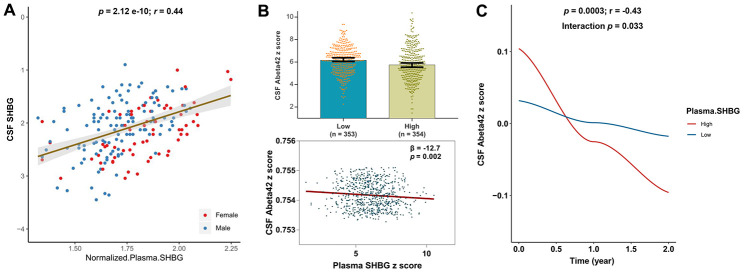
**Relationships of plasma SHBG with CSF SHBG and CSF Aβ42.** Plasma and CSF levels of SHBG were highly correlated (**A**). Higher levels of plasma SHBG were associated with lower levels of CSF Aβ42 after adjusting for age, gender, education, *APOE*4 status, and MMSE at baseline (**B**); Higher levels of plasma SHBG were associated with faster decline of CSF Aβ42 after adjusting for age, gender, education, *APOE*4 status, and diagnosis at baseline (**C**).

Data of SHBG levels both in plasma and in CSF were available for 188 subjects from ADNI-1. The correlation analysis showed that SHBG levels in plasma and CSF were highly related (p = 2.12 × 10 ^-10^, r = 0.44) (Figure 1A).

### Plasma SHBG was associated with lower CSF Aβ42

CSF biomarker abnormality is deemed as the earliest change during the AD course. The cross-sectional analyses revealed that plasma SHBG was negatively correlated with CSF levels of Aβ42 (r = -0.17, p = 0.0001). After adjusting for age, gender, education, *APOE4* status, and MMSE scores at baseline, a significant association was found between plasma SHBG and CSF Aβ42 (p < 0.005) (Figure 1B). No interactions with gender or *APOE*4 status were found, though the subgroup analyses indicated that the association was significant only in *APOE*4 non-carriers (p < 0.005). Longitudinally, higher plasma SHBG was associated with a faster decline in CSF Aβ42 (Figure 1C), after adjusting for age, gender, education, *APOE4* status, and clinical diagnosis. No significant associations were revealed for CSF tau proteins (Supplementary Table 1), though potential interactions with *APOE*4 status were found (Supplementary Table 2).

### Plasma SHBG accelerates hippocampus atrophy and decline in brain metabolism

**Figure 2:**
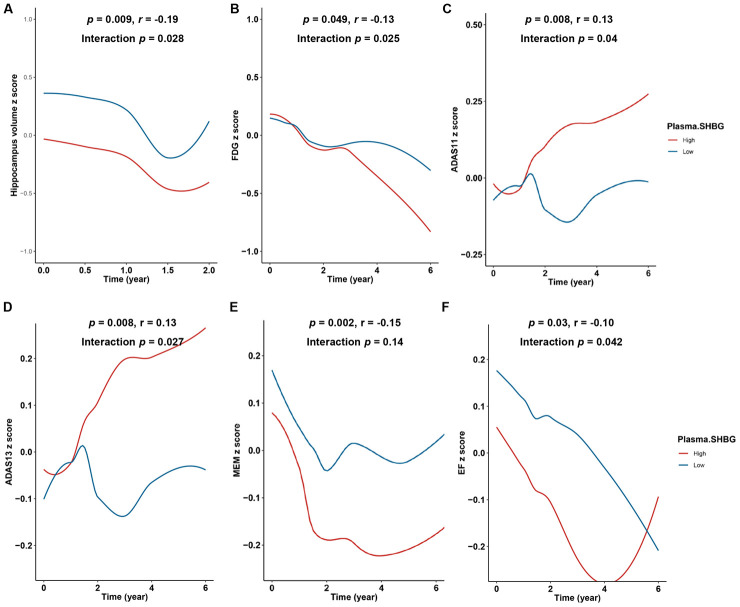
**Relationships of plasma SHBG with hippocampus atrophy, brain metabolism decline, and cognitive decline.** Individuals with higher SHBG showed faster rate of hippocampus atrophy (**A**) and brain metabolism decline (**B**). Individuals with higher plasma SHBG levels exhibited faster decline in general cognition (**C** and **D**), memory function (**E**), and executive function (**F**). All models were adjusted for age, sex, education, *APOE4* status, and baseline diagnosis. Abbreviations: ADAS = Alzheimer’s Disease Assessment Scale; MEM = Memory function; EF = Executive function. The first p value with index ® indicates the relationships between baseline plasma SHBG and change rates of the above phenotypes over follow-up. The second p value represents association results from the linear mixed effects models.

In the prodromal stage of AD, changes in CSF biomarkers were followed by structural atrophy as well as brain metabolic decline. We found individuals with higher plasma SHBG showed faster hippocampus atrophy (p = 0.028, Figure 2A) and brain metabolism decline (p = 0.025, Figure 2B). No significant interaction terms were found and sensitivity analyses barely changed the results.

### Plasma SHBG contributes to cognitive decline

With the accumulation of biomarker and imaging abnormalities, cognitive impairments become the major manifestations as AD progresses. We found that higher plasma SHBG levels conferred a faster decline in general cognition (Figure 2C and 2D), memory function (Figure 2E), and executive function (Figure 2F). Sensitivity analyses excluding those who developed AD within one year since baseline or adding TT and FTI as covariates barely changed the results.

### Plasma SHBG increases risk of AD dementia

**Figure 3:**
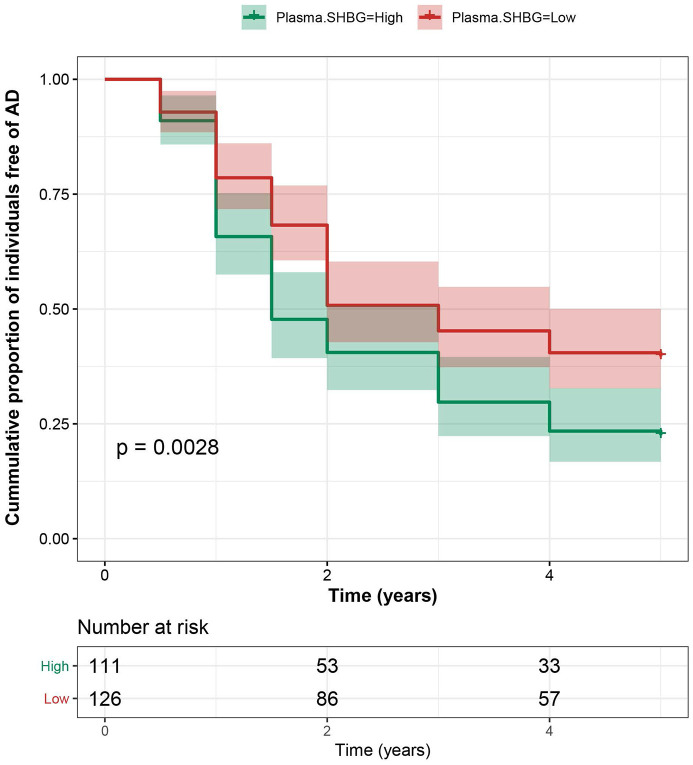
**Association between plasma SHBG and AD risk.** Subjects with higher plasma SHBG had an increased risk of developing AD, independent of age, sex, education, *APOE* genotype, and diagnosis.

In the ADNI cohort for incident AD dementia, 199 subjects were lost to follow-up and 237 were finally included (with a follow-up duration of 3.2 ± 2.4 years), among whom 164 subjects (36%) developed AD dementia. No significant differences in age, sex, education, and plasma SHBG at baseline were found between those who completed the follow-up and those who were lost. Compared with the lowest tertile (T1), subjects with higher plasma SHBG were associated with an average of 50% (T2) or 60% (T3) increased risk of developing AD dementia, independent of age, sex, education, *APOE* genotype, and diagnosis. The significance barely changed after further adjusting for lifestyle factors and vascular risk factors. (Figure 3) Stratified analyses indicated that the influences of plasma SHBG levels on the risk of incident AD dementia were stronger in the male group and those of advanced age (Table 2). Sensitivity analyses excluding those who developed AD dementia within one year since baseline or adding TT and FTI as covariates did not change the results.

**Table 2:** Hazard ratios with corresponding 95% confidence intervals of the association of SHBG levels with risk of Alzheimer’s disease according to strata of age, sex, and BMI.

	Tertile*	**N (case/total)**	**Model 1^a^**		**Model 2^b^**		**Model 3^c^**	
			**HR (95% CI)**	**p value**	**HR (95% CI)**	**p value**	**HR (95% CI)**	**p value**
Total (n = 237)	T1 (reference)	50/89	1	…	1	…	1	…
	T2	55/77	**1.52 (1.04-2.24)**	**0.03**	**1.50 (1.01-2.21)**	**0.04**	**1.71 (1.08-2.72)**	**0.02**
	T3	57/71	**1.91 (1.31-2.80)**	**0.001**	**1.63 (1.07-2.47)**	**0.02**	1.48 (0.99-2.23)	0.06
Male (n = 157)	T1 (reference)	36/70	1	…	1	…	1	…
	T2	40/56	**1.71 (1.09-2.68)**	**0.02**	**1.65 (1.04-2.61)**	**0.03**	**1.63 (1.01-2.64)**	**0.04**
	T3	25/31	**2.31 (1.38-3.86)**	**0.001**	**2.10 (1.23-3.59)**	**0.01**	**2.05 (1.15-3.66)**	**0.02**
Female (n = 80)	T1 (reference)	14/19	1	…	1	…	1	…
	T2	15/21	1.04 (0.50-2.26)	0.92	1.28 (0.59-2.79)	0.54	0.83 (0.31-2.22)	0.72
	T3	32/40	1.18 (0.63-2.22)	0.60	1.19 (0.62-2.31)	0.60	1.86 (0.83-4.17)	0.13
Age < 75y (n = 127)	T1 (reference)	31/52	1	…	1	…	1	…
	T2	24/37	1.09 (0.64-1.86)	0.75	1.09 (0.63-1.88)	0.76	0.95 (0.52-1.74)	0.87
	T3	28/38	1.48 (0.89-2.47)	0.13	1.48 (0.81-2.68)	0.20	2.03 (0.91-4.52)	0.08
Age ≥ 75y (n = 110)	T1 (reference)	19/37	1	…	1	…	1	…
	T2	31/40	**2.13 (1.20-3.79)**	**0.01**	**2.28 (1.23-4.22)**	**0.01**	**2.28 (1.19-4.34)**	**0.01**
	T3	29/33	**2.65 (1.47-4.77)**	**0.001**	**2.05 (1.08-3.88)**	**0.03**	1.88 (0.94-3.78)	0.07
BMI < 26 kg/m2 (n = 122)	T1 (reference)	24/38	1	…	1	…	1	…
	T2	26/36	1.27 (0.73-2.21)	0.40	1.52 (0.84-2.75)	0.17	1.58 (0.81-3.06)	0.18
	T3	41/48	**1.79 (1.08-2.96)**	**0.03**	1.75 (0.96-3.17)	0.07	1.87 (0.99-3.56)	0.06
BMI ≥ 26 kg/m2 (n = 115)	T1 (reference)	26/51	1	…	1	…	1	…
	T2	29/41	**1.76 (1.03-2.99)**	**0.04**	**1.86 (1.05-3.31)**	**0.04**	1.84 (0.96-3.53)	0.07
	T3	16/23	1.71 (0.92-3.19)	0.09	1.52 (0.77-3.02)	0.23	2.08 (0.79-5.44)	0.14

*SHBG levels (nmol/L) were log10-transformed: T1 <1.67; T2 = 1.67-1.84; T3>1.84

^a^model 1 – crude HR with no covariates adjusted

^b^model2 – HR adjusted for age, gender, education, *APOE4* status, and diagnosis at baseline.

^c^model 3 –HR adjusted for model 1 + diabetes mellitus type 2, depression, body mass index, hypertension, hyperlipidemia, sleep disorder, stroke history, cardiovascular disease, alcohol abuse, hearing loss, current smoking, and cancer.

Abbreviations: HR: hazard ratios; CI: confidence intervals; BMI: body mass index.

### Plasma SHBG is not a clinical diagnostic biomarker for mild AD dementia

The combination of the biomarker profile and the clinical classification divided subjects into 12 different groups that are summarized in Supplementary Table 3. There were 35 healthy control (HC) and 180 mild AD dementia. No significant difference in plasma SHBG was found between HC and AD groups (p = 0.17, Supplementary Figure 1A). We also assessed the diagnostic accuracy of plasma SHBG to discriminate between AD and HC. To this regard, a receiver operating characteristic (ROC) curve analysis was undertaken. The area under the curve was 0.584 (95% CI 0.481-0.687, p = 0.12, Supplementary Figure 1B).

## DISCUSSION

The present study provided multiple lines of evidence supporting plasma SHBG as a promising biomarker for disease progression in prodromal AD. We found that higher levels of plasma SHBG were associated with decreased CSF Aβ42, brain metabolism decline, hippocampus atrophy, cognitive decline, and increased AD risk.

The mechanisms by which SHBG was involved in AD occurrence might be explained by the free hormone hypothesis [8]. SHBG is one of corticosteroid-binding globulins (CBG) that deliver sex hormones in plasma to the outside of cells where some steroids are detached, internalized into the target cells, and bound to the intracellular receptors [9]. It was hypothesized that the breach equilibrium between the bound and free states might disturb the normal neuroprotective function of sex steroids [8, 10]. The SHBG-bound fraction of hormone is considered to be non-bioactive and it is inhibited from playing roles in neuronal functioning, such as synaptic formation, turnover, and transmission. The loss of these neuroprotective processes might lead to brain structural and functional abnormalities, and finally the occurrence of AD. It was found that the physiological response of mice to corticosteroid was decreased when the CBG expression was genetically suppressed [11]. Thus, it could be postulated that plasma SHBG might passively or actively influence neuroprotective functioning of steroids, by which it contributed to neurodegeneration in prodromal AD [12, 13].

The strata effect of the association between plasma SHBG and AD is complex, for which the evidence set seemed far less robust. Although we did not find influences of age strata on the effect sizes, subjects in the ADNI study are majorly restricted to adults at late-life stage. A recent imaging study indicated that higher plasma SHBG levels were also significantly associated with lower total and parietal grey matter volumes in middle-aged men [14]. Future studies should explore whether the associations found here can also be generalized to middle-aged adults. In accordance with previous findings [15, 16], the levels of plasma SHBG were higher in females from the ADNI. However, we found females had significantly lower levels of plasma SHBG in younger adults from the CABLE. Given that ADNI female participants were older than those in the CABLE, the percentage of postmenopausal females or estrogen replacement therapy might explain the discrepancy found here (Supplementary Table 4). However, this hypothesis warrants further investigation in future studies given that no supporting analyses were conducted here.

In the past decades, the preventive effects of hormone replacement therapy on cognitive decline or dementia have been widely studied. Unfortunately, these trials ended up with little success. It is also noteworthy that previous randomized controlled trials (RCTs) tended to focus on the hormone *per se* and hardly considered transports of the hormone transporter (such as SHBG). More efforts are thus needed in the future to understand the pathophysiological roles of SHBG in AD occurrence, which might provide new clues for future trials to prevent AD.

Several limitations exist. First, the conclusion from some subgroup analyses, such as analysis of gender strata, is less robust due to the limited sample size, which thus should be further validated in larger-scale studies. Second, the attrition bias might influence the credibility of the longitudinal analyses. High-quality cohort studies are warranted to confirm these findings. Third, different platforms for measuring SHBG and AD biomarkers in both samples constrained our comparison. Also, different races preclude us from drawing strong conclusions based on cross-validation. Fourth, the measurements of plasma SHBG were run in singlicate, which might increase the risk of measurement bias. Finally, the diagnostic values of plasma SHBG in differentiating AD from controls warrant further investigation. We failed to reveal plasma SHBG as a diagnostic biomarker of AD. This might be due to that only mild dementia was included in ADNI, and the difference might be observed only in advanced stage of AD.

In conclusion, we provided preliminary evidence showing that plasma SHBG could serve as a promising predictor of disease progression in the early stage of AD. The values of plasma SHBG in diagnosis, prediction and prevention of AD are still to be addressed, especially in larger prospective studies in the future.

## MATERIALS AND METHODS

### Study participants

A total of 707 non-demented adults who were northern Han Chinese were derived from CABLE since 2017. CABLE is an ongoing large-scale study majorly focused on AD’s risk factors and biomarkers in Chinese Han population. Individuals were recruited at Qingdao Municipal Hospital, Shandong Province, China. All enrolled participants were Han Chinese aged between 40 to 90 years. The exclusion criteria include (1) central nervous system infection, head trauma, epilepsy, multiple sclerosis or other major neurological disorders; (2) major psychological disorders; (3) severe systemic diseases (e.g., malignant tumors); (4) family history of genetic diseases. All participants underwent clinical and neuropsychological assessments, biochemical testing, as well as bio-sample (blood and CSF sample) collection. Demographic information and medical history were collected via a structured questionnaire and an electronic medical record system. We used the Diagnostic and Statistical Manual of Mental Disorders, 4^th^ Edition (DSM-IV) criteria [17] to define dementia. Individuals living with dementia were not included in the present study. The CABLE was approved by the institutional review boards of Qingdao Municipal Hospital and written informed consent was obtained from all participants or their guardians according to the Declaration of Helsinki.

The longitudinal data used in the present study were downloaded from the ADNI database (adni.loni.usc.edu). As a multicenter study, ADNI is designed to develop clinical, imaging, genetic, and biochemical biomarkers for the early detection and tracking of AD. The participants are adults aged 55-90 years with normal cognition (NC), mild cognitive impairment (MCI), or mild AD. Further information can be found at http://www.adni-info.org/ and in previous reports [18–20]. For the longitudinal analysis, 800 participants were selected from ADNI-stage 1 (ADNI-1) cohort. Each participant underwent an in-person interview for general health and function at the time of study entry by a standard assessment, including medical history, physical and neurological examination, as well as neuropsychological batteries. Baseline data were collected from 2005-2007 and follow-up data were collected during evaluations at sequential intervals of approximately 12 months. The ADNI was approved by institutional review boards of all participating institutions, and written informed consent was obtained from all participants or their guardians according to the Declaration of Helsinki.

The plasma SHBG levels were measured in 567 ADNI-1 participants (112 AD, 58 CN, and 397 MCI). To lower the clinical heterogeneity, we only included the non-demented individuals (n = 455). We further excluded 7 outliers, for which SHBG measurements were located outside the ±3SD range. Finally, 448 non-demented participants who had both plasma SHBG measurements and baseline covariates were included. As for the AD risk cohort, 237 participants had completed follow-up examinations during a five-year follow-up.

### Measurements of SHBG in plasma and CSF

For CABLE and ADNI, plasma and CSF samples for each subject were obtained in the morning following an overnight fast. The time from collection to freezing was mostly within two hours. Experimenters were blind to the clinical information of samples. In CABLE, plasma SHBG levels were determined with the Human SHBG ELISA kit (abcam, UK) on the microplate reader (Thermo Scientific™ Multiskan™ MK3). Samples were diluted 100-fold (gradient dilution) and run in duplicate (the mean intra-batch coefficient of variation (CV) was 6.67%). In ADNI, SHBG concentrations were tested using multiplex immunoassay panel on the Luminex xMAP platform by Rules-Based Medicine. The samples were run in singlicate while quality controls were performed in duplicate. The mean inter-assay CV was controlled < 20%.

### Measurements of CSF AD biomarkers

In the CABLE, CSF was collected by lumbar puncture in 10 ml polypropylene tubes before being sent to the lab within 2 hours. CSF samples were centrifuged at 2000g for 10 minutes. The thaw/freezing cycle was limited not to surpass 2 times. Baseline CSF Aβ1-42, tau, and p-tau181 were determined with the ELISA kit (Innotest β-AMYLOID (1-42), hTAU-Ag, and PHOSPHO-TAU (181p); Fujirebio, Ghent, Belgium) on the microplate reader (Thermo Scientific™ Multiskan™ MK3). The within-batch precision values were <5% (4.8% for Aβ42, 4.6% for tau, and 2.4% for ptau181). The inter-batch CVs were < 15% (9% for Aβ42, 12.2% for tau, and 10.9% for ptau181). In the ADNI, CSF procedural protocols have been described previously [21]. In brief, baseline CSF Aβ42 was measured using the INNOBIA AlzBio3 immunoassay (Fujirebio, Belgium). The within-batch precision value was 5.1-7.8% for Aβ42.

### PET imaging

All ADNI subjects underwent PET scanning procedures to study cerebral glucose metabolism. Subjects were injected with a dose of 18F-FDG in the resting state in a quiet darkened room. All sites performed 3D data acquisition, provided images corrected for Compton scatter, and measured attenuation correction based upon “transmission” and “blank” scans for those systems having rod sources or by CT scan for those sites having a PET/CT scanner.

### MRI measurement of hippocampus

The process of MRI acquisition in the ADNI has been described elsewhere [22, 23]. In brief, ADNI MRIs were acquired at multiple sites with 1.5T GE, Philips, and Siemens MRI scanners using a magnetization prepared rapid acquisition gradient echo (MP-RAGE) sequence. Two high-resolution T1-weighted MRI scans were collected for each participant using a sagittal 3D MP-RAGE sequence with an approximate TR = 2400ms, minimum full TE, approximate TI = 1000ms, and approximate flip angle of 8 degrees. Scans were collected with a 24 cm field of view and an acquisition matrix of 192 x 192 x 166 (x, y, z dimensions), to yield a standard voxel size of 1.25 x 1.25 x 1.2 mm. Images were then reconstructed to give a 256 x 256 x 166 matrix and voxel size of approximately 1 x 1 x 1.2 mm.

### Cognitive measures and AD diagnosis

The Alzheimer’s Disease Assessment Scale (ADAS) - cognition section was used to represent the global cognition. The validated composite scores for executive and memory functions were developed by reviewing the neuropsychological batteries to identify items which could be considered indicators of these two domains [24, 25]. In brief, the indicators of executive functions include Category Fluency, WAIS-R Digit Symbol, Trails A & B, Digit Span Backwards, and clock drawing. The indicators of memory function include relevant items of the Rey Auditory Verbal Learning Test (RAVLT), ADAS-Cog, Logical Memory, and Mini-Mental State Examination (MMSE). The National Institute of Neurological and Communication Disorders/Alzheimer’s Disease and Related Disorders Association (NINCDS–ADRDA) criteria [26] were used for the diagnosis of probable AD.

### Statistical analyses

R version 3.5.1 and GraphPad Prism 7.00 software were used for statistical analyses and figure preparation. p < 0.05 was considered significant except where specifically noted. Chi-square tests (for categorical variables), one-way ANOVA (for continuous variables with normal distributions), and Kruskal-Wallis test (for continuous variables with skewed distributions) were used to compare the baseline demographic, clinical, and diagnostic characteristics.

In case of skewed distribution (Shapiro-Wilk test > 0.05) for dependent variable, transformation was performed to approximate a normal distribution via “car” package of R software. As plasma SHBG levels were measured using different methods in ADNI and CABLE, we cannot achieve mutual transformation for these data and thus cutoff is defined using the median level of plasma SHBG: > 46.74 nmol/L for CABLE and > 56.23 nmol/L for ADNI. As for CABLE, linear regression was used to explore the cross-sectional relationships of SHBG (high vs. low with cutoff = 46.74 nmol/L) with CSF Aβ42 or tau levels after adjusting for age, gender, education, *APOE4* status, and MMSE score. Interaction terms for gender (male vs. female) and *APOE4* status (positive vs. negative) were used to explore whether strata effect existed. In case of any potential interaction (p < 0.1), subgroup analysis was further performed. As for ADNI data, the longitudinal influences of plasma SHBG at baseline on CSF AD biomarkers, cognition, hippocampus volume, and brain metabolism were explored using linear mixed effects models adjusted for age, gender, education, *APOE4* status, and clinical diagnosis at baseline. The linear mixed effects models had random intercepts and slopes for time and an unstructured covariance matrix for the random effects and included the interaction between time (continuous) and plasma SHBG (high vs low) as a predictor. All outcome variables in linear mixed-effects models were standardized to z scores to facilitate comparisons between modalities. The “lm”, “nlme”, “ggplot2”, and “car” packages in R 3.4.3 software were used to perform the above analyses.

Finally, the influence of plasma SHBG on the risk of incident AD was explored using the time-dependent Cox proportional hazards models. The adjusted risk, expressed as hazard ratio (HR) and 95% confidence interval (CI), was estimated for the association between incident AD and plasma SHBG levels at baseline. Individuals who did not develop AD or who were lost to follow-up were censored at the time of their last evaluation. The dependent measure was time from entry into the cohort to AD diagnosis. To estimate the influence of those lost, the baseline characteristics of those lost to follow-up and those who completed the follow-up were compared. In the Cox proportional hazard models, three models were employed with or without covariates: 1) analyses were conducted without adjusted covariates; 2) we adjusted for age, gender, education, *APOE4* status, and diagnosis; 3) we added diabetes mellitus type 2 (DM2), current depression, body mass index (BMI), hypertension, hyperlipidemia, sleep disorder, stroke history, cardiovascular disease (CVD), alcohol abuse, hearing loss, current smoker, and cancer as covariates.

Multiple subgroup and sensitivity analyses were conducted. First, given the gender differences in factors regulating SHBG levels [27], we re-ran all analyses in men and women separately. Second, we tested the robustness of the results by excluding those who developed dementia whine one year since baseline. Third, we repeated all analyses after further adjusting for plasma levels of total testosterone (TT) or free testosterone index (FTI) which were calculated by dividing TT by SHBG.

Finally, to test whether plasma SHBG could also be a diagnostic biomarker, each ADNI participant was assigned to specific a group according to their A-T-N biomarker profile and clinical stage [28]. First, A-T-N profile defined whether a participant was exposed (positive or negative) to specific pathologic processes, including aggregated Aβ (A, as indicated by CSF Aβ_42_), aggregated tau (T, as indicated by CSF ptau_181p_), and neuronal injury (N, as indicated by CSF T-tau). Cutoff values used to define positive groups were CSF Aβ_42_ < 976.6 pg/ml for A+ group, CSF ptau_181p_> 21.8 pg/ml for T+ group, and CSF T-tau > 245 pg/ml for N+ group, respectively [29]. Herein, the CSF core biomarkers were measured using the electrochemiluminescence immunoassays Elecsys on a fully automated Elecsys cobas e 601 instrument (Roche). T and N group were merged together and TN+ indicated either T or N was abnormal. Second, participants were classified based on their clinical dementia rating (CDR) global score into cognitively unimpaired (CDR = 0), very mild dementia (CDR = 0.5), and mild dementia (CDR = 1.0). Next, a receiver operating characteristic (ROC) analysis was used to test the diagnostic value of plasma SHBG to discriminate AD subjects from controls. We computed the areas under the curve (AUC), and we tested whether they were significantly different from the null hypothesis that the AUC equals 0.50, which corresponds to a random test

### Ethics approval

CABLE was approved by institutional review boards of Qingdao Municipal Hospital and written informed consent was obtained from all participants or their guardians according to the Declaration of Helsinki. ADNI was approved by institutional review boards of all participating institutions, and written informed consent was obtained from all participants or their guardians according to the Declaration of Helsinki.

## Supplementary Material

Supplementary Figure 1

Supplementary Tables
