# Supplementary material for: Plasma sex hormone-binding globulin predicts neurodegeneration and clinical progression in prodromal Alzheimer's disease
Source: Aging (Albany NY). 2020 Jul 22;12(14):14528–41. doi: 10.18632/aging.103497 (PMC7425468; doi:10.18632/aging.103497)
Supplement: Supplementary Figure 1 [file aging-12-103497-s002..pdf]

SUPPLEMENTARY FIGURE

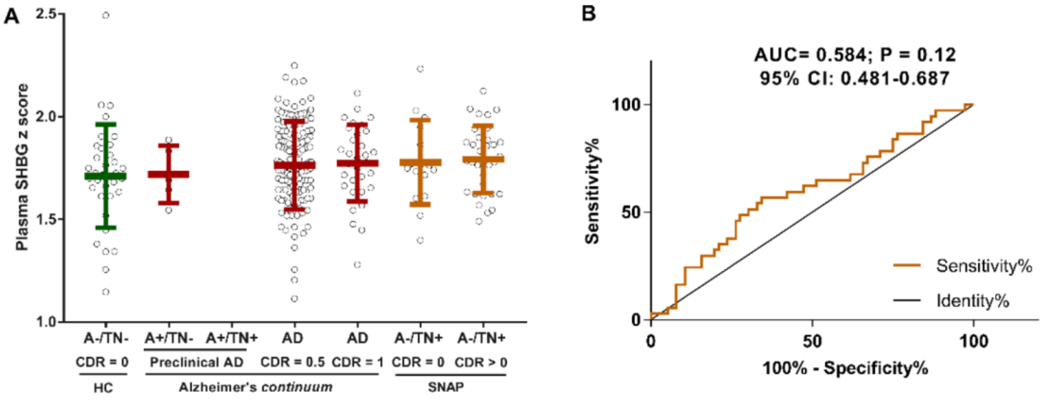

Supplementary Figure 1. Plasma SHBG is not a clinical diagnostic biomarker for mild AD dementia. No significant difference in plasm SHBG was found between HC and mild AD groups (**A**). We also assessed the diagnostic accuracy of plasma SHBG to discriminate between AD and HC. To this regard, a receiver operating characteristic (ROC) curve analysis was undertaken. The area under the curve was 0.584 (95% CI 0.481-0.687, p = 0.12, **B**).
