# Supplementary material for: Plasma sex hormone-binding globulin predicts neurodegeneration and clinical progression in prodromal Alzheimer's disease
Source: Aging (Albany NY). 2020 Jul 22;12(14):14528–41. doi: 10.18632/aging.103497 (PMC7425468; doi:10.18632/aging.103497)
Supplement: Supplementary Tables [file aging-12-103497-s001..pdf]

SUPPLEMENTARY TABLES

Supplementary Table 1. Associations between plasma SHBG and CSF AD biomarkers in CABLE.

CSF biomarker	Continuous				Categorical			
	beta	*p* value [&]	interaction with APOE4	interaction with Gender	beta	*p* value [&]	interaction with APOE4	interaction with Gender
**abeta42**	-2.07E-06	**0.00021**	0.76	0.73	-5.55E-05	**0.00255**	0.35	0.24
tau	-5.68E-05	0.212	**0.16**	0.72	-1.77E-03	0.2379	0.24	0.84
ptau	-1.03E-05	0.28156	**0.07**	0.3	-2.12E-04	0.5004	**0.14**	0.59
**tau/abeta42**	1.72E-03	**0.02547**	0.27	0.75	4.69E-02	0.063395	**0.15**	0.44
**ptau/abeta42**	9.62E-04	**0.00238**	**0.16**	0.31	2.74E-02	**0.00868**	**0.07**	0.13

[&] Chi-square tests (for categorical variables), Student t test (for continuous variables with normal distributions), and Mann-Whitney U test (for variables with skewed distributions) were used to compare demographic, clinical, and diagnostic variables.

Supplementary Table 2. Subgroup analyses according to APOE4 status in CABLE.

Subgroup	Apoe4+				Apoe4-			
CSF biomarker	Continuous		Categorical		Continuous		Categorical	
	beta	*p* value [&]	beta	*p* value	beta	*p* value [&]	beta	*p* value
**abeta42**	-1.96E-06	0.113	-1.78E-05	0.683	-2.03E-06	**0.00129**	-6.11E-05	**0.00275**
tau	3.14E-05	0.77326	2.00E-03	0.60024	-8.48E-05	0.0933	-2.60E-03	0.111
ptau	1.72E-05	0.42	1.15E-03	0.12028	-1.75E-05	0.103	-4.85E-04	0.161
tau/abeta42	2.35E-03	0.23683	3.84E-02	0.58107	1.38E-03	0.1003	4.51E-02	0.09579
**ptau/abeta42**	1.33E-03	0.10128	2.61E-02	0.3597	7.99E-04	**0.0207**	2.60E-02	**0.0196**

[&] Chi-square tests (for categorical variables), Student t test (for continuous variables with normal distributions), and Mann-Whitney U test (for variables with skewed distributions) were used to compare demographic, clinical, and diagnostic variables.

Supplementary Table 3. Classification of ADNI participants based on their biomarker profile and clinical stage.

<table>
<tr><td colspan="2" rowspan="2"></td><td colspan="3">Clinical stage（C）</td></tr>
<tr><td>CDR = 0
(cognitively unimpaired)</td><td>CDR = 0.5
(very mild dementia)</td><td>CDR = 1
(mild dementia)</td></tr>
<tr><td rowspan="5">Biomarker profile</td><td>A-/TN-</td><td>Healthy controls
n = 35</td><td>n = 25</td><td>n = 1</td></tr>
<tr><td>A+/TN-</td><td>Preclinical AD A+/TN-
n = 5</td><td>n = 31</td><td>n = 6</td></tr>
<tr><td>A+/TN+</td><td>Preclinical AD A+/TN+
n = 0</td><td>AD CDR = 0.5
n = 148</td><td>AD CDR = 1
n = 32</td></tr>
<tr><td rowspan="2">A-/TN+</td><td>n = 16</td><td>n = 30</td><td>n = 3</td></tr>
<tr><td colspan="3">Suspected non-Alzheimer’s pathophysiology (SNAP)</td></tr>
</table>

A, amyloid-b biomarker status; AD, Alzheimer’s disease; CDR, clinical dementia rating; N, neurodegeneration biomarker status; T, tau pathology biomarker status. ADNI participants were classified based on their CSF biomarker profile and their clinical stage, which yielded 12 different categories. Columns depict the clinical stage (C) as defined by the clinical dementia rating (CDR) scale. Rows depict the biomarker profiles. Each of the three biomarker groups (A/T/N) was binarized into positive or negative (+/). T and N were merged to simplify the classification: TN indicates that both T and N biomarkers are normal, and TN+ indicates that T and/or N biomarkers are abnormal. The grey highlighting indicates the grouping used for comparisons in the main text. Light grey highlights the healthy controls (n = 38), middle grey the groups included in the Alzheimer’s continuum (n = 205) and dark grey the suspected non-Alzheimer’s pathophysiology (SNAP) group (n = 40). Bold text indicates the groups analysed in the main analysis, namely “healthy controls”, “Preclinical AD A+/TN ”, “Preclinical AD A+/TN+”, “AD CDR = 0.5” and “AD CDR = 1”.

Supplementary Table 4. Comparison of the female characteristics between CABLE and ADNI.

	CABLE	ADNI	P value
Age (mean ± SD, year)	63.4 ± 11.2	74.0 ± 7.3	< 0.0001
SHBG (mean ± SD, nmol/L)	48.7 ± 26.7	72.6 ± 32	< 0.0001
